# Biliary Roadblocks: A Case of a Short Cystic Duct in Routine Gallbladder Surgery

**DOI:** 10.7759/cureus.71201

**Published:** 2024-10-10

**Authors:** Ethan Dimock, Forrest Bohler, Alise Haddad, Ibrahim Baida

**Affiliations:** 1 Medical School, Oakland University William Beaumont School of Medicine, Auburn Hills, USA; 2 General Surgery, Corewell Health William Beaumont University Hospital, Royal Oak, USA

**Keywords:** difficult laparoscopic cholecystectomy, general surgery, hepato-pancreato-biliary surgery, laparoscopic cholecystectomy complication, short cystic duct

## Abstract

This case report describes the intraoperative finding of a short cystic duct in a 73-year-old female patient undergoing a laparoscopic cholecystectomy. This case highlights the importance of careful preoperative imaging and intraoperative techniques to minimize complications when anatomical variations such as a short cystic duct are present. The report also underscores the necessity of heightened awareness for variations in biliary anatomy, as well as the potential risks they pose during cholecystectomy procedures.

## Introduction

Laparoscopic cholecystectomy remains the gold standard treatment for symptomatic cholelithiasis. However, anatomic variations of the biliary tree can complicate this seemingly routine procedure, significantly increasing the risk of bile duct injury or other complications. One such variation is a short cystic duct, which may predispose patients to difficulties in identification and isolation during surgery.

Anatomical variations of the cystic duct, including short or absent ducts, have been shown to occur in approximately 4-10% of patients undergoing cholecystectomy [1]. Such variations often complicate the dissection of Calot's triangle and can increase the risk of complications such as bile leaks, ductal injuries, and inadvertent common bile duct exploration. Additionally, patients with short cystic ducts had a higher rate of bile duct injuries compared to those with normal anatomy, underscoring the importance of careful dissection and the use of intraoperative imaging techniques [1].

## Case presentation

**Figure 1:**
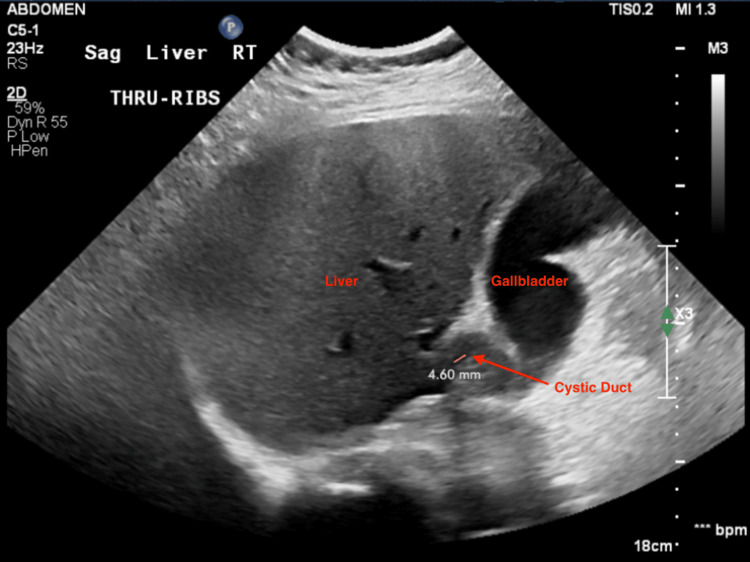
Right upper quadrant ultrasound, displaying the short cystic duct (indicated by a red arrow)

The patient we present is a 73-year-old Caucasian female who initially presented to the emergency department (ED) in a hospital outside of Detroit, Michigan, United States, complaining of generalized abdominal pain, nausea, and vomiting. Her symptoms began shortly after receiving an epidural injection for chronic back pain from her pain management physician. An ultrasound (Figure 1) of the right upper quadrant (RUQ) demonstrated the presence of gallstones and a moderate amount of sludge within a mildly distended gallbladder, as well as a short cystic duct measuring 0.46 cm.

The patient was taken to the operating room and administered general endotracheal anesthesia. She was positioned, prepped, and draped in the usual sterile fashion. An incision was made above the umbilicus, and an open Hassan cannula was placed without difficulty. Additional 5 mm ports were placed in the RUQ under direct visualization. The gallbladder was retracted to expose the cystic structures, and the hepatocystic triangle was identified. During dissection, it became apparent that the patient had an abnormally short cystic duct. The cystic duct was carefully dissected free at the level of the infundibulocystic junction to ensure no compromise of the common bile duct. Both the cystic duct and artery were controlled with clips. The gallbladder was then removed from the liver bed using an electrocautery hook, placed in a specimen bag, and removed from the abdominal cavity without difficulty. The abdominal wall fascia was closed with heavy Vicryl sutures, and the skin was closed with Monocryl and Histocryl sutures.

Despite the case's complexity, the surgery was completed without complications, though the short cystic duct increased the technical difficulty. Postoperatively, the patient recovered well and was discharged on postoperative day 3.

## Discussion

Variations in biliary anatomy can present significant challenges during cholecystectomy procedures. The average adult cystic duct is 2-4 cm in length, and a short cystic duct is defined as a duct that is less than 0.5 cm in length [1]. In a 2016 study that looked at anatomical variations of the cystic duct, 198 patients who underwent a magnetic resonance cholangiopancreatography (MRCP) were studied, and it was found that only 1% of them had a short cystic duct [1]. The short cystic duct, as encountered in this case, is a well-documented but rare anatomical variation, and its presence can significantly increase the risk of bile duct injury during surgery. The most notable increase in risk occurs during laparoscopic cholecystectomies [2]. Studies have emphasized the need for thorough preoperative planning, particularly in patients with a history of complex medical conditions and previous abdominal surgeries, as these factors may obscure the normal anatomy or contribute to postoperative complications [2,3].

One of the most popular intraoperative strategies for reducing the incidence of bile duct injuries in laparoscopic cholecystectomies is the critical view of safety (CVS)" technique [4]. This technique involves three steps: clearing the hepatocystic triangle of tissue, separating the lower third of the gallbladder from the liver, and ensuring that the gallbladder is connected to only two structures, the cystic artery and the cystic duct.

Intraoperative cholangiography is another valuable tool for preventing bile duct injury in cases where the anatomy is unclear. In one study conducted in 2023, the use of cholangiography significantly reduced the incidence of major bile duct injury during cholecystectomy, especially in cases involving anatomical variations such as short cystic ducts [5]. In the case we present, cholangiography was not utilized.

Complications from a short cystic duct are not limited to bile duct injury. There is also an increased risk of hemorrhage during dissection, particularly if the cystic artery is inadvertently damaged [6]. In addition, the proximity of the cystic duct to the common bile duct in such cases increases the difficulty of clipping and dividing the duct. In this case, the use of surgical clips to secure the cystic duct was successful, though caution was exercised due to the proximity of surrounding structures.

## Conclusions

This case highlights the challenges associated with the presence of a short cystic duct during laparoscopic cholecystectomy. Careful preoperative imaging and the use of careful intraoperative dissection were crucial in avoiding complications. Surgeons should remain vigilant for anatomical variations, particularly in patients with complex medical histories, and should be prepared to adjust their surgical approach accordingly. Understanding the potential risks associated with a short cystic duct can help optimize patient outcomes and reduce the likelihood of intraoperative complications.
